# The changing landscape of care: does ethics education have a new role to play in health practice?

**DOI:** 10.1186/s12910-015-0005-0

**Published:** 2015-05-08

**Authors:** Julie Wintrup

**Affiliations:** Centre for Innovation and Leadership in Health Sciences, University of Southampton, Highfield Campus, Southampton, SO17 1BJ UK

**Keywords:** Healthcare education, Ethics education, Professional ethics, Moral agency, Ethical self-formation

## Abstract

**Background:**

In the UK, higher education and health care providers share responsibility for educating the workforce. The challenges facing health practice also face health education and as educators we are implicated, by the way we design curricula and through students’ experiences and their stories.

This paper asks whether ethics education has a new role to play, in a context of major organisational change, a global and national austerity agenda and the ramifications of disturbing reports of failures in care. It asks: how would it be different if equal amounts of attention were given to the conditions in which health decisions are made, if the ethics of organisational and policy decisions were examined, and if guiding collaborations with patients and others who use services informed ethics education and its processes?

**Discussion:**

This is in three parts. In part one an example from an inspection report is used to question the ways in which clinical events are decontextualised and constructed for different purposes. Ramifications of a decision are reflected upon and a case made for different kinds of allegiances to be developed. In part two I go on to broaden the scope of ethics education and make a case for beginning with the messy realities of practice rather than with overarching moral theories. The importance of power in ethical practice is introduced, and in part three the need for greater political and personal awareness is proposed as a condition of moral agency.

**Summary:**

This paper proposes that ethics education has a new contribution to make, in supporting and promoting ethical practice – as it is defined in and by the everyday actions and decisions of practitioners and people who need health services. Ethics education that promotes moral agency, rather than problem solving approaches, would explore not only clinical problems, but also the difficult and contested arenas in which they occur. It would seek multiple perspectives and would begin with places and people, and their priorities. It would support students to locate their practice in imperfect global contexts, and to understand how individual and collective forms of power can influence healthcare quality.

## Background

This paper explores how ethics education in professional health programmes contributes to safe, effective and ethical practice. It suggests ways in which educators might more directly support and promote the ethical self-formation of students across disciplines.

A greater focus on new professional responsibilities and self-awareness is argued for, given the changing organisation of health and social care services in the UK as many more providers join what has been described as a ‘marketplace’. The impact of economic decisions also requires a response by health professionals, who have a role in minimising their effect on patients and on the quality of care and treatment. Finally the undermining effect of high profile inquiries into the neglect and mistreatment of vulnerable people by healthcare professionals is considered in light of the need to rebuild trust and confidence.

There is much work to be done. The attention on health services and the way we educate the workforce following the Francis report offers an opportunity to reflect on the meaning of ethical practice, and how it needs to promote more effectively the goal of high quality healthcare.

In part one I discuss how problematic constructions of interdisciplinary and patient/carer relationships influence healthcare and serve to create or perpetuate disciplinary divisions. The case is made for a more nuanced appreciation of the way cultural and organisational influences prevent more unifying forms of problem analysis, and I go on to make a case for more and different allegiances. In part two the attention being paid to the personal characteristics of healthcare workers is problematised in light of critiques of the ‘values’ agenda. A more politically informed analysis is proposed. Part three introduces the need for an enhanced and confident sense of moral agency, and considers ways in which the idea of ethical self-formation has a contribution to make. Connections are made between the intimate and immediate work of healthcare, and general trends that influence the way we view ourselves as professionals. New contributions by students to global communities of practice that offer inspiring and energising visions of practice are seen as supporting a reflexive stance.

Finally the argument is outlined in brief for broadening and complicating the work to be done within professional ethics education, if it is to offer a critique of healthcare’s more fundamental problems.

A necessarily brief and speculative introduction to such ideas is offered here in keeping with the scope of a journal paper. Its purpose is to generate reflection, discussion and collaboration among those interested in the education and practices of the health workforce, and in quality healthcare.

## Discussion

### New allegiances

In 2011 a press release by the Care Quality Commission (CQC) reported that a doctor in a National Health Service hospital prescribed water to patients who were at risk of dehydrating [1]. The report stated that other staff agreed to the practice because it ‘worked’ and that the ward was short staffed. A subsequent newspaper commented “nurses sometimes left patients so thirsty that the only way for doctors to ensure they had enough liquid was to add ‘drinking water’ to medication charts” [2].

I would like to reflect briefly on this as a way of thinking about some of the moral complexities of a particular situation, and how interdisciplinary relationships are viewed. Firstly, the doctor in question clearly wished to improve patients’ health. Viewed as a doctor-patient interaction, and through more traditional ethical perspectives, the doctor’s actions were caring and - in many ways – constituted an effective or pragmatic response to a difficult situation; by treating drinking water as medication, patients no longer risked dehydration. However the need to ‘prescribe’ water indicated a much broader set of ethical concerns on the part of the doctor. Considering the relationships *between* nurses, doctors, and patients (rather than merely thinking about nurses, doctors, or patients as individuals) gives a stronger sense of the mutually connected dimensions of such a decision, and the ways in which both doctors and nurses – and inevitably many other health professionals - were implicated in sustaining these unsatisfactory conditions.

The newspapers appear happy to conclude that the nurses involved were fundamentally uncaring. However research has shown that short staffing within a particular ward is more often a cause of ‘care left undone’ [3]. If such underlying causes were a feature of the doctor’s decision, prescribing water perhaps unintentionally perpetuated and even legitimised wider organisational deficiencies. In terms of team working and collaborative patient care, the general acceptance that ‘it worked’ indicates a degree of passivity or compliance.

Of course, speculating on an actual event is problematic and risks misconstruing or denying the deeply situated nature of moral problems. But we already know a good deal about the importance of relationships, within and amongst disciplines and across the entire community of practice, in particular with patients themselves and those who care about them. The Bristol Royal Infirmary inquiry [4] highlighted how distant, task-oriented relationships between professionals, managers, patients and relatives negatively influenced care and treatment. Personal and professional distances noted between nurses, doctors and managers in particular reflect other inquiry reports over many years [5]. Sir Robert Francis was criticised for blaming primarily front line staff rather than senior managers, who, it has been claimed, were complicit in the maintenance of the inadequate treatment regimes at Mid Staffordshire [6].

Poor standards of care also reflect ineffective safety and governance mechanisms. While the last resort of whistleblowing remains available to staff, such high-risk activity is known to be very personally costly [7]. Even diligent and caring staff must fear the ramifications of speaking out or reporting colleagues. Such fears may lead people to alleviate the worst effects of dysfunctional situations rather than to challenge them. This is within contexts in which a powerful need to belong has been reported in nurse education [8] and complex social games have been found to determine medical career chances [9]. Much has also been reported on the toxic effect of bullying cultures, their influence on individual behaviours, and the ways in which emotionally unsafe environments perpetuate fear and silence [10].

Moral strain - highly associated with feelings of helplessness and work-related stress - is found to exist among practitioners working with inadequate resources who feel the need to compromise personal standards [11]. ‘Human factors’ research highlights just how dangerous tiredness, inattention and fear can be and how errors rise when a blame culture is evident [12]. Twigg [13] provides a stark reminder of the low status accorded to physical care work and its delegation to the least powerful and most precariously employed members of the workforce. Add to this the overwhelmingly female composition of the direct-care workforce and it is easy to see why Colley [14] surfaces the potential for exploitation of those socialised to take responsibility for caring work.

Professionals operate, to use Holland’s ([15]: page 155) words, ‘in a distinctive ethical sphere [that] places distinctive normative requirements on [them]’. In other words, they are required to adhere to and promote a set of rules and standards regardless of the wider environment - even when the conditions of their work impede or prevent their being able to do so. The CQC account allows ethical problems beyond the individual professional-patient relationship to be explored, bringing interdisciplinary relationships and obligations, manifestations of power and compliance, fear, cultural influences and corporate responsibilities within the domain of ethical practice and therefore, I argue, of ethics education.

Interprofessional education has been part of undergraduate health programmes for many years and offers an early opportunity to learn about what Emmerich calls ethico-political problems [16]. It is most likely to be when priorities differ that teams most need to work together, yet are at most risk of returning to a discipline-focussed default position. Equally, the participation of patients and people using services or caring for others offers a powerful contribution to ethics education across the entire educational journey, including the lifelong Continuing Professional Development (CPD) of experienced practitioners. When allegiances across boundaries develop and become a new norm, negotiated objectives and more rigorous decision making strategies necessarily become an everyday feature of professional work.

### Power is real

Francis called for a ‘safer, committed and compassionate and caring service’ ([17], page 1416), urging staff to ‘put patients before themselves’ and to ‘be honest and open with patients regardless of the consequences for themselves’. These invocations paved the way for the Chief Nursing Officer’s so-called 6Cs – Compassion, Commitment, Caring, Courage, Competence and Communication [18].

While this may provide a useful mnemonic, this conception of ‘values’ is out of step with other important work in the area. For instance, Sackett ([19], page 1) has looked beyond the values of the professionals, to ‘the unique preferences, concerns and expectations each patient brings to the clinical encounter’. In a similar vein, Valenti et al. [20] offer a thoughtful analysis of how compulsorily detained patients’ values differ from those of staff, offering better ways to understand and manage ethical conflicts. Frameworks for studying conflicting values, including organisational conflicts and vested interests, came out of the work of Fulford et al. [21].

Health services are deeply affected by political and ‘macro-ethical’ [22] issues such as financial and resourcing decisions, contractual arrangements, and workforce changes. Yet despite Kennedy’s [4] assertion that government-driven ‘top down’ change presents a risk to safe care and treatment, these topics are unlikely to find their way into ethics courses in health professionals’ education, or - because of how the scope of practice is defined – even thought to be relevant.

As educators, whether primarily in practice or academic roles, we have a responsibility to more thoroughly prepare healthcare students for the realities of professional practice in an increasingly mixed economy of care with its bureaucracy, visibility, increasing forms of delegation, marketisation and complex regulatory and governance arrangements. Lines previously drawn around what is considered to be ‘ethical practice’ may need to be redrawn to include a more astute understanding of moral responsibility and delegation in such uncertain and rapidly changing contexts.

If educational messages are focussed primarily on ‘shared values’ or ‘individual personal qualities’ we risk ignoring the far more complicated interplay between people, policy directives, political ambitions and health outcomes. These phenomena directly affect how people *act*, and set certain limits on possible courses of action. Students – and experienced practitioners learning through CPD – might usefully be supported to learn more about and to question these broader structures and contexts.

A recent study that involved professionals ‘mapping’ their knowledge of older people’s admission routes into hospital, to learn how unnecessary admissions might be avoided [23], offers a critique of discipline-focussed approaches. Practitioners’ deep knowledge of parts of the service contrasted with partial or impressionistic understandings of other parts. The likelihood of a multi-professional/multi-agency response – to work more effectively across boundaries or to recognise and challenge contradictions in policy enactment - is much reduced in such circumstances. However ‘good’ the quality of care in narrow or specialist areas, and however caring the individual practitioner, practice that requires ‘boundary-crossing’ risks falling short of being ethical in this broader sense. Knowledge and influence beyond single parts of the system and, importantly, on policy development and implementation, is necessary if ethico-political awareness is to develop [16].

What to include in ethics education in professional programmes, and its nature more generally, has been the subject of much debate; criticism has been made of its tendency to develop ‘problem solvers’ while ignoring the development of sensitivity and judgement [24]. Lawlor [25] has argued that abstract moral theories are often too difficult to apply in meaningful ways to real clinical problems during undergraduate education. Overarching moral theories constitute what the philosopher Raymond Geuss [26] has called the ‘ethics first’ approach. A political philosopher, Geuss argues against beginning with abstract notions of ‘fairness’ or ‘justice’ then moving from such abstract concepts to make normative political claims about what governmental policy ought to do (for instance). Instead he advocates starting from real situations, historically and socially located, in order to understand politics. While this anti-theoretical stance is a very different project to our task of educating professionals – who are of course bound by professional standards – the idea of beginning with ‘real’ practice offers new questions and rejects the imposition of ideological concepts which might bear no relation to students’ experiences of healthcare practices. His criticism of these ‘ethics first’ approaches is that they fail to think about how and why we act in particular situations and deny the importance of power, when ‘it is advisable to pay attention to it’ ([26], page 97). By suspending, albeit experimentally, the overarching value claims made by health leaders, we might usefully look more thoughtfully at *how* and *where* health problems are managed, at *who helps* people live with illness or disability, and at the ways in which health professionals and others contribute most effectively (or fail to do so).

Understanding how power manifests in healthcare relationships is intrinsic to ethical practice. The more nuanced work around values understands power to be inherent within relationships. By its nature it is interested in when values and interests conflict, and the ways in which power can be unequally distributed.

In similar vein, the principles that inform regulatory codes and other normative frameworks have a dual purpose, not only to guide and shape professional identity formation, but to identify when an individual falls below a standard [27]. This is fundamental to professions’ ability to exert power over individuals in order to self-regulate and control membership. Being ‘struck off’ a professional register is career ending, yet paradoxically fear of such a possibility can lead to professional self-interest taking priority over patients’ interests.

Understanding the power of organisations, formal and informal groups and teams, and how to utilise forms of power, is part of developing a sense of agency that extends beyond the individual. Such agency is described by Beckett as social, embodied, negotiated and performative in nature [28]. Kramer’s [29] study showed how developing the ability to influence events, through greater understanding, knowledge and confidence, reduced the distress and conflict experienced by novices. A sense of being able to shape and influence events, with and alongside others, emerges as invigorating in exactly the same way that feeling helpless, isolated and pawn-like leads to listlessness and passivity. Viewed in this way, advocating on the part of a patient, or their family member or a work colleague, is not an act of ‘compassion’, but an act of solidarity.

### Moral agency and ethical self-formation

The renewed emphasis on values and the personal characteristics of health professionals has been accompanied by plans to further embed and measure certain characteristics within healthcare university programmes. Smajdor [30] presents a compelling case against this project, seeing as a flawed endeavour the aim to ‘incentivise’ such emotional responses as compassion, because doing so risks encouraging cynical forms of compliance. Research into moral development supports her scepticism. It is unsurprising that students – who are able to identify the socially desirable responses in written tests that seek to examine their moral imaginations and ethical principles - often fail to enact the principles that they espouse. In one study, those scoring highest on moral reasoning tests also scored highest for ‘amorality’ [31]. Furthermore, exposure to the vagaries of practice has been found to reduce students’ moral standards and expectations [32] or leave them more confused about what constitutes ‘right’ and ‘wrong’ forms of action [33].

While certain personal qualities are necessary to practise effectively - such as a concern for people and an interest in their doing well - these are generally intrinsically motivated. Seeking to elicit them using extrinsic motivators, for instrumental purposes, is likely to have perverse effects.

As a source of learning, then, inquiry processes can reveal just how difficult allocating moral responsibility can be, especially when poor communication, inadequate levels of training or supervision, and confusion around delegated responsibilities muddy the water. These kinds of inquiry reports can show us the ways in which accountability is contingent – in sharp contrast to the reassuring learning outcomes that package and describe ethics courses in higher education.

For students to feel confident in this more situated and reflective form of moral agency, their *own* values, beliefs and sense of identity need to be the starting point for developing as professionals. The sociologist Anthony Giddens ([34], page 86) suggests that in the modern world people are called upon to engage in ‘reflexive projects of the self’, where they pursue certain projects, styles, and modes of inhabiting the world. Giddens’ notion can help us to think about students’ reflexive projects of the self, and how ethics education might speak to them in this more intimate way.

To focus on ethical theories is to leave unanswered the question most often raised by students: what am I supposed to *do* if an issue is not clear-cut? Being told that every situation is unique, and that ‘it depends’, can feel like short change – especially if students have shared personal stories, concerns and feelings. Worse, it may even be interpreted as anything goes, or imply that ‘ethics’ as a topic is irrelevant to life.

In contrast, learning about healthcare work in more general, global terms – the uncertainties, injustices, frustrations, as well as the successes and rewards – enables a professional, personal and collective identity formation that is influenced and altered in important ways by such knowledge. Giddens ([34]: 86) offers an example of what he calls the reflexive shaping of self-identity: “A black woman heading a single-parent household, however constricted and arduous her life, will nevertheless know about factors altering the position of women in general, and her own activities will almost certainly be modified by that knowledge”.

In the same way, trying to analyse messy and partial clinical experiences in an ethics session can be frustrating and leave students feeling as if they have failed to convey *exactly* what was happening or explain why they are left with particular feelings [35]. Whereas viewing such experiences in light of the position of healthcare professionals in general – the effects of moral strain or the excitement of witnessing a successful intervention - can be helpful in connecting one’s own endeavours to those of colleagues, to wider trends or to special interest groups. In this way, students’ use of blogs and other kinds of social media can be viewed as reflexive and identity forming pursuits, that position and connect them as people in the world, with interests and membership of communities within and outside their healthcare work. However such collegiality needs to extend to a commitment to act with and alongside others if the rhetoric of the heroic individual is to be challenged.

Moral agency can be a fragile and fleeting experience, in need of active reinforcement if it is not to be subsumed by ideologies imposed from elsewhere. When student nurses and care workers blew the whistle on cruel practices taking place at Garlands Hospital in 1996, an internal inquiry criticised their lack of understanding of how practice in the ‘real world’ operated (and also blamed “the type of patient in this ward” as Butler and Drakeford [5] found to characterise so many inquiries) [36]. Some knowledge and understanding of how corrupt, micro-political forces were diminishing strategic governance at that particular Trust through those years [37], would have helped them to remain resilient and view themselves as moral agents, rather than as victims of an unjust system.

Ethical self-formation, described by Giddens [32] as a reflexive project of the self, offers us a way of thinking about how *education* – through its social and relational forms of learning - might combat the ‘existential isolation … from the moral resources necessary to live a full and satisfying existence’ [32, page 9]. Such a sense of self is *actively shaped* by events over time and yet has an enduring dimension because of the self-conscious and reflexive ways in which persons shape their sense of who they are and how they act upon the world. Understanding how different forms of power operate both to *conceal*, in ways described by Epstein [38], and to *sustain* such injustices offers a more constructive way of using distressing experiences as catalysts for action.

## Summary

The central argument of the paper is that ethical practice requires health professionals to be effective moral agents. A more responsible form of moral agency, I want to suggest, requires an understanding of power: how it manifests in relationships, organisational cultures and structurally, and the ways in which it introduces risk and threatens safe practice. Such agency requires a sense of oneself, as intimately caught up in and influential upon the lives and wellbeing of others, as a part of communities and with interests and allegiances that transcend disciplinary and patient/professional boundaries. Educators themselves, then, need a better understanding of the complexities of practice and its changing nature, in order to counteract the prevailing discourses that place compassion and other personal emotions and characteristics at the forefront of educational requirements.

Firstly I argue that ethics education needs to reflect the many constituencies and perspectives, in order to prepare students for collaborative work - across disciplines, and with people who use health services as patients and carers. This is imperative given the increasingly complicated arenas in which healthcare takes place, with changing responsibilities and types of delegation in a more mixed economy of care.

Next, I contend that ethical practice takes place in politically informed arenas in which power is a key feature of actions and decisions. Examining the nuances and complexities of why people act in certain ways is pertinent to understanding the boundaries of ethics and to extending them to include broader motives and plans. It is central to the case for change that while ethics education focuses predominantly on the more theoretical and abstract elements of moral philosophy, or promotes an ethics first approach, an opportunity is lost to view these messier, difficult and shared problems as moral ones that can be challenged.

Finally I suggest that ethics education has a role to play in supporting students to be fully involved as moral agents, influencing and being shaped by communities and individuals within society beyond the narrow clinical domains. This is in contrast to the contemporary project to inculcate particular values through healthcare education, with its risk of instilling a narrow and unsophisticated understanding of what constitutes ethical practice.

It is for this reason that the stance of the educator is proposed as pivotal to the success of such alternative approaches. Appreciation of the unique and situated ethical problems students encounter requires openness and a preparedness to engage with uncertainty. It requires a concerted commitment to improvement that crosses traditional demarcations and unifies, understanding such responses to promote resilience and to be more effective and sustainable than heroic individualism. It requires a willingness to explore new ideas, and to rehearse viewpoints, and ultimately to accept that confusion can be creative and is essential to transformative learning. Ethics education, conceived of in these ways, might look different and is likely to take place outside as well as inside the classroom. Interprofessional education and collaborative learning with patients and others offer ways to begin re-imagining how it might more directly support ethical practice.
